# Supplementary material for: Contracting the private health sector in Thailand’s Universal Health Coverage
Source: PLOS Glob Public Health. 2023 Apr 28;3(4):e0000799. doi: 10.1371/journal.pgph.0000799 (PMC10146570; doi:10.1371/journal.pgph.0000799)
Supplement: Attachment — Submitted filename: Response to reviewers .docx [file pgph.0000799.s001.docx]

Reviewer #1:

Major Corrections:

The study has some serious shortcomings which must be resolved.

1. The findings seems to be largely based on the document review, and the analysis of indepth interviews is insignificant. The authors must clearly specify the purpose of the indepth interviews and present its analysis and finding in a distinct manner.

**RESPONSES**

Many thanks for the observation. We had further analysed the verbatim transcription of the key informant interviews. We had fully responded and introduced the following new sub-section 3.4 and two others from purchaser and provider perspective.

3.4 Effectiveness of the Current Private Engagement

3.4.1 Purchaser Perspective

3.4.2 Private Sector Provider Perspective

2. The conclusions drawn from the study does not align with the analyses and findings. The study fails to establish how contracting with the private healthcare facilities has filled the gap between demand and supply of the "quality care". Authors must include required evidence in the analysis to support their conclusions.

**RESPONSES**

The discussion section was overhauled and fully rewritten with reference to key literatures on contracting.

3. The text is repetitive at many places. The authors must revise the manuscript to make it more succinct and to the point.

**RESPONSES**

Many thanks for the comments, we have thorough reviewed revised as suggested.

**Minor Corrections:**

Page-5 Line-6: There is typographical error the word "proving" make in sense in the statement.

**RESPONSES**

Thank you for the comment, the typo is replaced by “providing”

Page-16 Line-8 The line should be rephrased to make it clearer.

**RESPONSES**

Many thanks, we have clarified as followed:

Of the 274 private providers, 193 facilities serve as both primary care units and main contracting units which entered into a contractual relationship with NHSO, 78 facilities accepted referrals from main contracting units

Page-16 Line-11 & 12 are repetition of text in line 3& 4.

**RESPONSES**

Thank you for the comment. The text “Note that some facilities register with NHSO as more than one category type. Hence sum of three types of facilities exceeds 274 facilities” was removed.

Page-16 Line-14-12 The paragraph gives contradicting information. Line 15 tells 100 bed as minimum resource and standard requirements for all type of facilities but the line 18 say 150 beds for private facilities.

**RESPONSES**

SSO defines criteria for being a main contracting unit as having at least 100 beds and 12 specialties. The following sentence which says, “Private main contractors are usually equivalent to the size of provincial hospitals with a 150-500 bed capacity” can create mis understanding. We mean MOPH provincial hospitals have between 150 and 500 bed.

To prevent mis-understanding the text “Private main contractors are usually equivalent to the size of Ministry of Public Health provincial hospitals, which have 150-500 bed capacity” was removed.

Page-18 Section 3.2 It is informed that NHSO can contract all three categories but "In practice NHSO primarily contracts with Main Contracting Units". The reason for such an anomaly should be explained to give readers more insights into the challenges of contracting.

**RESPONSES**

We remove the following text as it creates misunderstanding.

~~NHSO can undergo contractual agreements with all three categories of service providers (as described in section 2), with direct payment mechanisms established for the contracted facilities.~~

We remove the text

~~In practice NHSO primarily contracts with Main Contracting Units;~~

And replace by the following:

However, NHSO may not make a direct contract with primary care units and leave primary care provision in responsibilities of the Main Contracting Units. NHSO applies this practice to most health centers belong to Ministry of Public Health (MOPH) in all 76 provinces outside Bangkok. It leaves MOPH’s district hospitals to manage service and financial agreements with their own subsidiary health centers

Page-19 Section 3.3 Data on the ratio of applications and selected contracting unit will be helpful for the reader to understand the openness of competition and process efficiency of contracting.

**RESPONSES**

Thank you for the observation; which is important to assess how competitive the private sector market in providing services to UCS and SHI.

We have got back to SSO and NHSO’s responsible persons, number of application data are internal document and not kept systematically and that they feel reluctant to share. Only the selected contracts are announced regularly in Royal Gazette, see <http://www.ratchakitcha.soc.go.th/DATA/PDF/2565/E/001/T_0001.PDF>

Page-22 Line-8 The word "Kind" might have different meanings in different contexts. It should be specified and made clear with the use of examples in the context of study.

**RESPONSES**

Many thanks for the suggestion. We clarify the issue on in kind payment to health care providers as followed

For example, NHSO authorizes Government Pharmaceutical Organization to purchase peritoneal dialysis solution; contracts Thai Post Distribution to deliver these solutions to patients’ home nation-wide; pays Main Contracting Units where patients registered with for supervising and supporting patients at home.

Page-23 Line-10 The idea of "relative weight" used for payments may be hard to understand for global readers. It should be elaborated briefly.

**RESPONSES**

Thank you for the suggestion. At the beginning of that paragraph, we clarify the relative weight as followed:

The DRG relative weight is the relative resource intensity of each inpatient. It is the ratio between average resource for one DRG and total resource for all DRG. Each disease group was assigned with a relative weight; the higher the DRG weight, the more resources are required to treat a patient, meaning the more payment to the providers. Thailand had applied DRG systems for more than two decades.

Page-23 Line-18 The text should be rephrased to make it comprehensible.

**RESPONSES**

Thank you for the suggestion, we had fully elaborated and clarified the complex payment methods applied by SSO. The whole paragraph was removed and replaced by the following.

To incentivize providers to provide adequate treatment for out-patients with chronic diseases, an additional 453 THB per registered SHI member for each main contractor hospital was offered. Half of this amount is paid on a monthly basis for 11 months calculated from the number of points per capita member earned by providing outpatient services for the 26 chronic diseases by each contractor. On the last payment in month 12th, the contractor hospitals receive the remaining half of 453 Baht based on its points per capita earned as a share of total national points per capita by all contractors in a year.

Further, certain equipment such as orthopedic prosthesis can be reimbursed using a fee-schedule. SSO also pays an additional 80% of the amount of expense over 1,000,000 THB for high-cost in-patient service of more than a million Bath. This complex mix of additional payment method applied by SSO aims to incentivize adequate treatment for more complex and high-cost outpatient and inpatients.

Page-23 Line 19-21 The idea that Supra- and sub-contractors deal with main contracting agency for all the purposes and not SSO is in repetition. This repetition should be removed.

**RESPONSES**

The text related to sub- and supra-contractor was removed.

Page-25 Line 3-7 Grammatical errors in the sentence. Check the usage of verb "compliance".

**RESPONSES**

Thank you for the observation. We replace the sentence by the following:

Step one accreditation was granted for facilities which established preventative measures and risk identification systems in place

Page-26 Line 1 The sentence should be rephrased to remove repetition of words and clarity.

**RESPONSES**

Many thanks for the suggestion, we edited as followed:

In fiscal year 2007, NHSO earmarked a budget of 0.76 Baht per capita member (35.7 million Baht in total) as additional incentives based on the achievement of different accreditation status.

Reviewer #2

This is a comprehensive description of two purchasers’ approaches to contracting out with private hospitals and clinics in Thailand within its universal health coverage system. Using descriptive case study methods, the study provides detailed insights into the

Major comments.

1. The authors could better situate this study within the broader literature, specifically on strategic purchasing – and the way in which contracts are a component of strategic purchasing to achieve health system objectives.

Some key papers seem to be missing, e.g., this paper from Thailand, https://www.ncbi.nlm.nih.gov/pmc/articles/PMC6706967/#R9

And this paper from SSA may also be worth reviewing - https://www.tandfonline.com/doi/full/10.1080/23288604.2022.2051795

If the authors could provide a summary of this broader literature on strategic purchasing in the introduction, and then again in the discussion, this would help to interpret the findings, and the limitations and challenges with the contracting models in Thailand.

**RESPONSES**

Many thanks for the suggestion, at the end of first paragraph of Background section, we introduced the following text with two references.

An approach of purchasing organizations ‘to find the best intervention to purchase, providers to purchase from and the best payment mechanisms and contracting arrangement to pay for such interventions’ is a primary aspect of strategic purchasing. Strategic purchasing involves organizational relationships between four key actors: government, purchasers, providers, and patients. Between purchasers and providers, the key policy objective is to improve provider performance for which a country will have its own set of governance structure shaped by its underlying values of the country’s health and political systems. Despite its importance, strategic purchasing function has been neglected in pursuing UHC in low- and middle-income countries.

2. It would also help to introduce the discussion of fraud earlier on in the study. Could the authors clarify why the case study methods didn’t include a description of the incentives or mitigation strategies against fraudulent behavior or other aspects of poor performance.

**RESPONSES**

Many thanks for the suggestion. A new sub-section 3.3.6 “Enforcement of Contract Through Audit” was introduced.

NHSO pioneered three categories of audit. First, coding audit is used to detect upcoding of DRG relative weight, DRG creep, which is a deliberative process to gain higher reimbursement [36]. DRG claims, randomly selected or targeted samples of outliers, were retrospectively reviewed by trained independent auditors. Overclaimed amounts were asked to return to NHSO otherwise legal actions would be taken in several cases. Second, billing audit identifies outstanding or abnormal claims by health facilities for services paid outside capitation or DRG such as Pap Smear for cervical cancer. By 2021, total 2272 auditors were trained and actively function. Though SSO does not develop auditing system, it applies NHSO coding audit though not systematic and regularly.

SSO has never terminated contracts though poor performing facilities voluntarily leave the Scheme. Prior to 2019, NHSO has terminated a few private contractors in Bangkok due to fraud and failed quality standard. In the past, NHSO only pressured the poor performance not to renew the contract.

3. The authors appears to be biased in favor of contracting private providers, and perhaps they could provide more nuanced presentation of the strengths and challenges drawing on the theoretical and empirical literature. E.g., this statement is overly strong and the empirical basis is limited – “contracting private providers are effective and should be advocated in developing countries.”

**RESPONSES**

We agree that the statement “contracting private providers are effective and should be advocated in developing countries” is a bit strong. We removed and also the discussion section was rewritten.
